# Systematic selections and forensic application evaluations of 111 individual identification SNPs in the Chinese Inner Mongolia Manchu group

**DOI:** 10.3389/fgene.2022.944580

**Published:** 2022-09-05

**Authors:** Congying Zhao, Hui Xu, Yating Fang, Ming Zhao, Qiong Lan, Man Chen, Shuyan Mei, Bofeng Zhu

**Affiliations:** ^1^ Guangzhou Key Laboratory of Forensic Multi-Omics for Precision Identification, School of Forensic Medicine, Southern Medical University, Guangzhou, China; ^2^ Microbiome Medicine Center, Department of Laboratory Medicine, Zhujiang Hospital, Southern Medical University, Guangzhou, China; ^3^ Key Laboratory of Shaanxi Province for Craniofacial Precision Medicine Research, College of Stomatology, Xi’an Jiaotong University, Xi’an, China

**Keywords:** individual identification, SNP, massively parallel sequencing, Chinese Inner Mongolia Manchu group, forensic genetics

## Abstract

Single nucleotide polymorphism (SNP) possesses a promising application in forensic individual identification due to its wide distribution in the human genome and the ability to carry out the genotyping of degraded biological samples by designing short amplicons. Some commonly used individual identification SNPs are less polymorphic in East Asian populations. In order to improve the individual identification efficiencies in East Asian populations, SNP genetic markers with relatively higher polymorphisms were selected from the 1,000 Genome Project phase III database in East Asian populations. A total of 111 individual identification SNPs (II-SNPs) with the observed heterozygosity values greater than 0.4 were screened in East Asian populations, and then, the forensic efficiencies of these selected SNPs were also evaluated in Chinese Inner Mongolia Manchu group. The observed heterozygosity and power of discrimination values at 111 II-SNPs in the Inner Mongolia Manchu group ranged from 0.4011 to 0.7005, and 0.5620 to 0.8025, respectively, and the average value of polymorphism information content was greater than 0.3978. The cumulative match probability and combined probability of exclusion values at II-SNPs were 7.447E^-51^ and 1-4.17E^-12^ in the Inner Mongolia Manchu group, respectively. The accumulative efficiency results indicated that the set of II-SNPs could be used as a potential tool for forensic individual identification and parentage testing in the Manchu group. The sequencing depths ranged from 781× to 12374×. And the mean allele count ratio and noise level were 0.8672 and 0.0041, respectively. The sequencing results indicated that the SNP genetic marker detection based on the massively parallel sequencing technology for SNP genetic markers had high sequencing performance and could meet the sequencing requirements of II-SNPs in the studied group.

## Introduction

Individual identification is one of the important tasks of forensic genetics, which is of great significance for a criminal investigation to identify the individual traceability of the biological material collected from the crime scene. However, due to the limitations of a smaller number of genetic markers and longer fragments of amplicons, short tandem repeat (STR) genetic markers are difficult to use in the genotyping of degraded biological samples (Kidd et al., 2006; Sanchez et al., 2006). Therefore, single nucleotide polymorphism (SNP) could be used as an effective supplementary tool to the existing STR genetic markers; it provides more clues in the forensic identification and case litigation stages because of their low mutation rates (10^-8^) (Amorim and Pereira, 2005), ability to be designed for short amplicons and wide distributions in the genome.

In 1998, a human SNP genetic map containing 2227 SNP genetic markers was first reported by WANG et al. (1998). Since then, databases with SNP data including the dbSNP, HapMap, and Human Genome Diversity Project (HGDP), have been built by different countries and institutions. Through these public databases, some scholars have screened out the different SNPs and constructed a variety of multiple amplification systems for different forensic research purposes in recent years. In forensic genetics, the Kidd laboratory proposed the criteria for selected individual identification SNP (II-SNP) genetic markers in 2006 (Kidd et al., 2006). To date, 90 autosomal SNP genetic markers from the Precision ID Identity Panel, and 94 autosomal SNP genetic markers from the ForenSeq™ DNA Signature Prep kit are commonly used in forensic practices (Guo et al., 2016; Li et al., 2018; Sun et al., 2019; Tao et al., 2021).

However, due to the allelic frequency distributional differentiations among different continental populations, it is uncommon for some given SNPs to have high heterozygosites in different populations around the world. At present, several SNPs, including rs251934, rs1886510, rs740910, rs729172, and rs2056277, in the common kit were reported to show poor forensic efficiencies in the East Asian populations (Zhang et al., 2015). Therefore, the selection of II-SNPs suitable for East Asian populations become the focus of current researchers nowadays.

The detection and genotype methods of SNP genetic markers include single-strand conformation polymorphism (Doi et al., 2004), denaturing gradient gel electrophoresis, DNA chip (Doi et al., 2004), SNaPshot (Fondevila et al., 2017), and massively parallel sequencing (MPS) (de la Puente et al., 2017), and so on. Among them, SNaPshot technology is a minisequencing technology that is the most commonly used method in forensic DNA laboratories, but it only accommodates a limited number of SNPs and reduces the forensic effectiveness of a multiplex system using SNP genetic markers (Daniel et al., 2015). In recent years, MPS technology has emerged as an ideal method for SNP genetic markers due to the virtues of containing more SNPs, reducing the risk of contamination, and progressively reducing detection cost.

According to the China Statistical Yearbook 2021 compiled by the National Bureau of Statistics of China (http://www.stats.gov.cn/tjsj/ndsj/2021/indexch.htm), the population of Manchu (1,0423,303) ranks the fifth largest among Chinese ethnic minorities, which are mainly distributed in the Inner Mongolia Autonomous Region, Jilin, Beijing, and other provinces. Among them, the Inner Mongolia Autonomous Region with ethnic minorities stretches from northeast to southwest in a long and narrow shape in the northern border area of China. The unique geographical locations as well as the wide distributions of ethnic minorities make it one of the long-standing and necessary regions in population genetic differentiation research. Nowadays, most of the Manchu groups living in the Inner Mongolia Autonomous Region are the descendants of the Manchurian eight banners, who were stationed in Guihua and Suiyuan cities (both of which belong to today’s Hohhot area) in the Qing dynasty (Adnan et al., 2018). With the end of the Qing regime, the Manchu banner people were disbanded and gradually formed mixed situations with Mongolian and Han populations. Previous population genetic research of the Manchu group mainly stemmed from the limited forensic genetic markers, including autosomal STRs (He and Guo, 2013), X-chromosome STRs (Xing et al., 2019), Y-chromosome STRs (Adnan et al., 2018), and mitochondrial DNA (mtDNA) (Zhao et al., 2011), but so far, there have been few reported SNP data from the Manchu group .

In this study, we systematically selected and preliminarily assessed the forensic efficacies of autosomal 111 II-SNPs to increase the individual identification efficiencies of SNPs in East Asian populations. Further forensic efficacy validation and population comparisons were investigated in the Chinese Inner Mongolia Manchu (IMM) group and other reference populations.

## Materials and methods

### Selection criteria of SNP genetic markers

SNPs with relatively higher polymorphisms in East Asian populations were selected from the 1000 Genomes Project phase Ⅲ database (Sherry et al., 2001). The Han Chinese in Bejing (CHB), Southern Han Chinese (CHS), Chinese Dai in Xishuangbanna (CDX), Kinh in Ho Chi Minh City (KHV), and Japanese in Tokyo (JPT) were merged as the East Asian populations. The specific selection criteria were as follows: 1. II-SNPs conform to the Hardy–Weinberg equilibrium (HWE) and linkage equilibrium; 2. II-SNPs should locate on different chromosomes, or the physical distances among any two II-SNPs on the same chromosome are greater than 10Mb; 3. The allele frequencies of bi-allelic II-SNPs range from 0.4 to 0.6 and the minor allele frequencies of multi-allelic II-SNPs are greater than 0.01; 4. These II-SNPs have not been found to be associated with any diseases; 5. The observed heterozygosity (Ho) values of II-SNPs in East Asian populations are greater than 0.4.

### Sample collection and processing

The 187 bloodstain samples were collected from Chinese Inner Mongolia Manchu individuals. All volunteers stated that they were healthy and unrelated within three generations, then signed the written consent forms. This experiment was approved by the ethics committees of the Xi’an Jiaotong University Health Science Center and Southern Medical University (Approval No. 2019-1,039). The 26 populations from the 1000 Genomes Project phase Ⅲ database were included as reference populations. The detailed information on populations are listed in Supplementary Table S1. First, a 1.0 mm bloodstain sample was taken from the center of the dried blood spot and placed in a centrifugal tube and then incubated at 60°C after adding 25 μL clean buffer; second, the centrifuge tube was taken out after brief centrifugation, and 20 μL supernatant was discarded after quick blowing for 3-5 times; third, 95 μL of clean buffer was added again, and then 90 μL supernatant was discarded; and finally, the remaining part was used as a DNA template.

### Library preparation, sequencing, and data analysis

The first-round PCR reaction mixture was mixed with 25 μL PCR Enzyme Mix, 0.5 μL PCR Clean Enzyme, 4 μL PCR Primer Pool, and DNA template. The PCR cycle parameters were listed as follows: 12 cycles at 95°C for 15s, 60°C for 5 min, 72°C for 30s, and a final extension at 72°C for 2 min. Then the first-round PCR products were purified by DNA Clean Beads (MGI Tech, Shenzhen, China).

The second-round PCR reaction mixture included 25 μL PCR Enzyme Mix, 0.5 μL PCR Clean Enzyme, 1 μL PCR Additive, and 2 μL PCR Primer A. The PCR cycle parameters were listed as follows: 16 cycles at 95°C for 15s, 60°C for 2 min, 72°C for 30s, and a final extension at 72°C for 2 min. The second-round PCR products were purified in the same way as in the previous step.

The purified PCR products were quantified by the Qubit^®^ dsDNA HS Assay kit (Thermo Fisher Scientific, MA, USA). Then the single-chain cyclization reaction solution and enzyme digestion reaction solution were prepared to cyclize and digest the library, respectively. DNA nano balls were prepared by adding 5 ng digested product and elution buffer up to 20 μL.

DNA sequencing was based on the MGISEQ-2000RS (MGI, Shenzhen, China), and the sequencing parameter was set to ‘FCL SE50+10’. The quality control and preprocessing of sequencing data were conducted by SOAPnuke software (Chen et al., 2018). The filtered sequencing data were aligned with the hs37d5 reference genome by the Burrows-Wheeler Aligner (BWA) software (Li and Durbin, 2010). Finally, the results of SNP calling were obtained by FreeBayes (https://github.com/freebayes/freebayes) based on the Bayes algorithm.

### Statistical analyses

The MPS performances at 111 II-SNPs in the IMM group were evaluated by three parameters: depth of coverage (DoC), allele coverage ratio (ACR), and noise level (NL) values. The box plots of DoC, ACR, and NL values were produced by Origin v2021 software. The *p* values of the HWE exact tests and linkage disequilibrium (LD) analyses were calculated by Genepop v4.0 software (ROUSSET, 2008). The allele frequency (AF), polymorphism information content (PIC), and expected heterozygosity (He) values of 111 II-SNPs in the IMM group were estimated by the PowerStats v1.2 software. The match probability (PM), power of discrimination (PD), and power of exclusion (PE) values of 111 II-SNPs in the IMM group were analyzed by STRAF online software (Gouy and Zieger, 2017) (http://www.cmpg.iee.unibe.ch/services/shiny/index_eng.html). The forensic parameters of 111 II-SNPs in the IMM group were visualized by the heatmap using TBtools V1.0 software (Chen et al., 2020). The forensic identifications of the selected 111 II-SNPs in complex kinship tests (full sibling and half sibling) were simulated by Familias v3 software (Kling et al., 2014). To further explore the population genetic differences among the IMM group and reference populations, genetic distances (*D*_*A*_ distances) and pairwise fixation index (*F*_*ST*_) values among the IMM group and 26 reference populations were calculated by the DISPAN software and Arlequin V3.5 software, respectively (EXCOFFIER and LISCHER, 2010). Phylogenetic analysis based on *D*_*A*_ distance values among the IMM group and 26 reference populations was performed by Evolview V3 software (Subramanian et al., 2019). The principal component analysis (PCA) at the individual level was performed by Origin software (https://www.originlab.com) based on the original SNP genotyping data of the IMM group and 26 reference populations. The population genetic structure analysis of 27 populations was conducted by the STRUCTURE V2.3.4 software (Porras-Hurtado et al., 2013) based on the Bayesian algorithm, and the optimal *K* value was determined by STRUCTURE Harvester software (Earl and VonHoldt, 2012).

## Results

### Preliminary evaluations of 111 screened II-SNPs in East Asian populations

**FIGURE 1:**
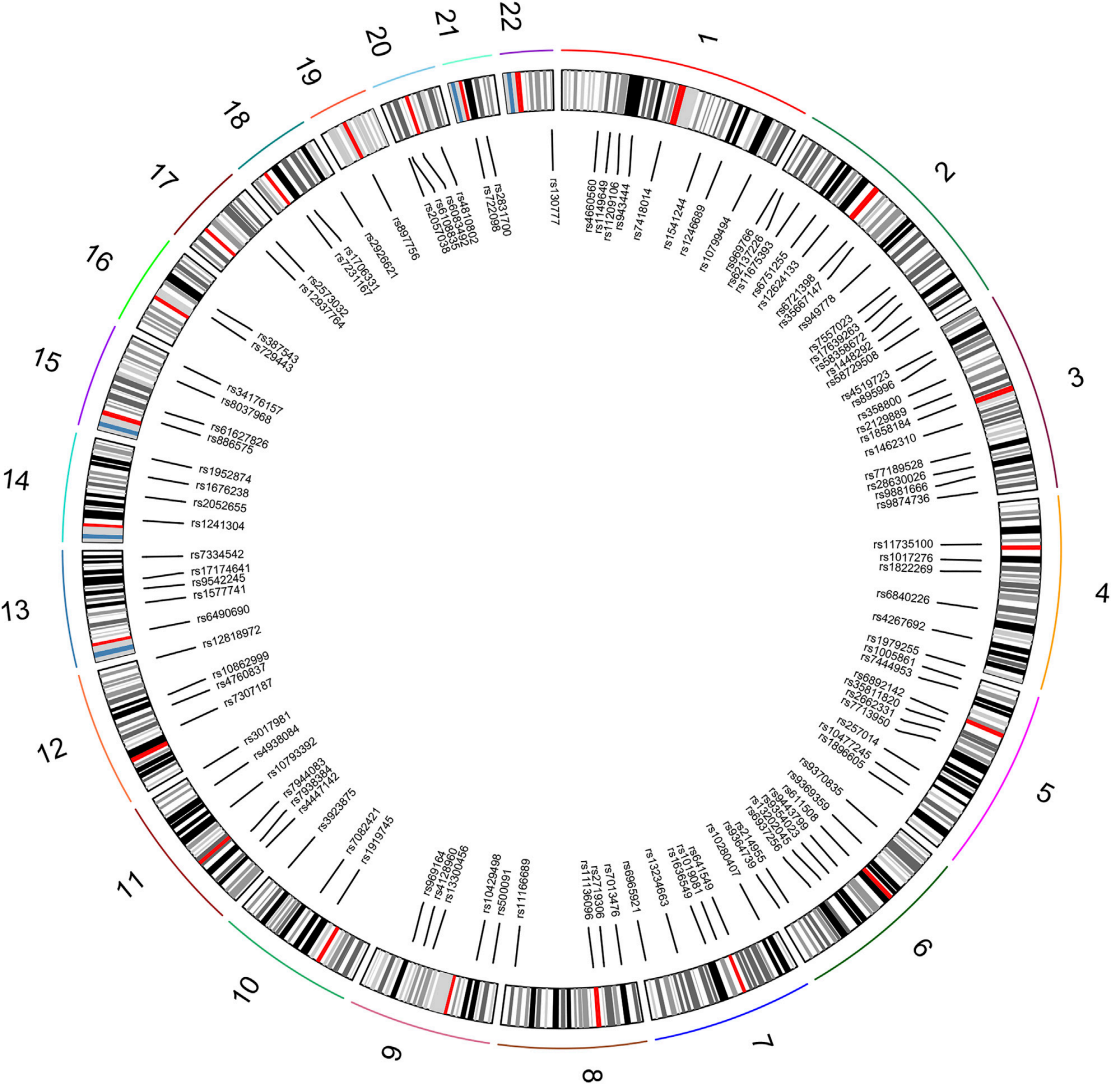
Distributions of 111 II-SNPs on different autosomes.

A total of 111 autosomal II-SNPs, including 22 multi-allelic SNPs and 89 bi-allelic SNPs, were screened after layers of filtering. All 111 loci were located on 22 pairs of autosomes, with 1–13 loci on each autosome. The SNP distributions on the chromosome were displayed and listed in Figure 1 and Supplementary Table S2, respectively. The results showed that the average Ho, PIC, and PD values of 111 II-SNPs were 0.5317, 0.4013, and 0.6397, respectively. The cumulative PM and PE values were 7.15E^-51^ and 1-9.96E^-13^, respectively, which indicated that these 111 II-SNPs showed relatively higher forensic application potential in East Asian populations.

### Sequencing performances of 111 II-SNPs in the IMM group

**FIGURE 2:**
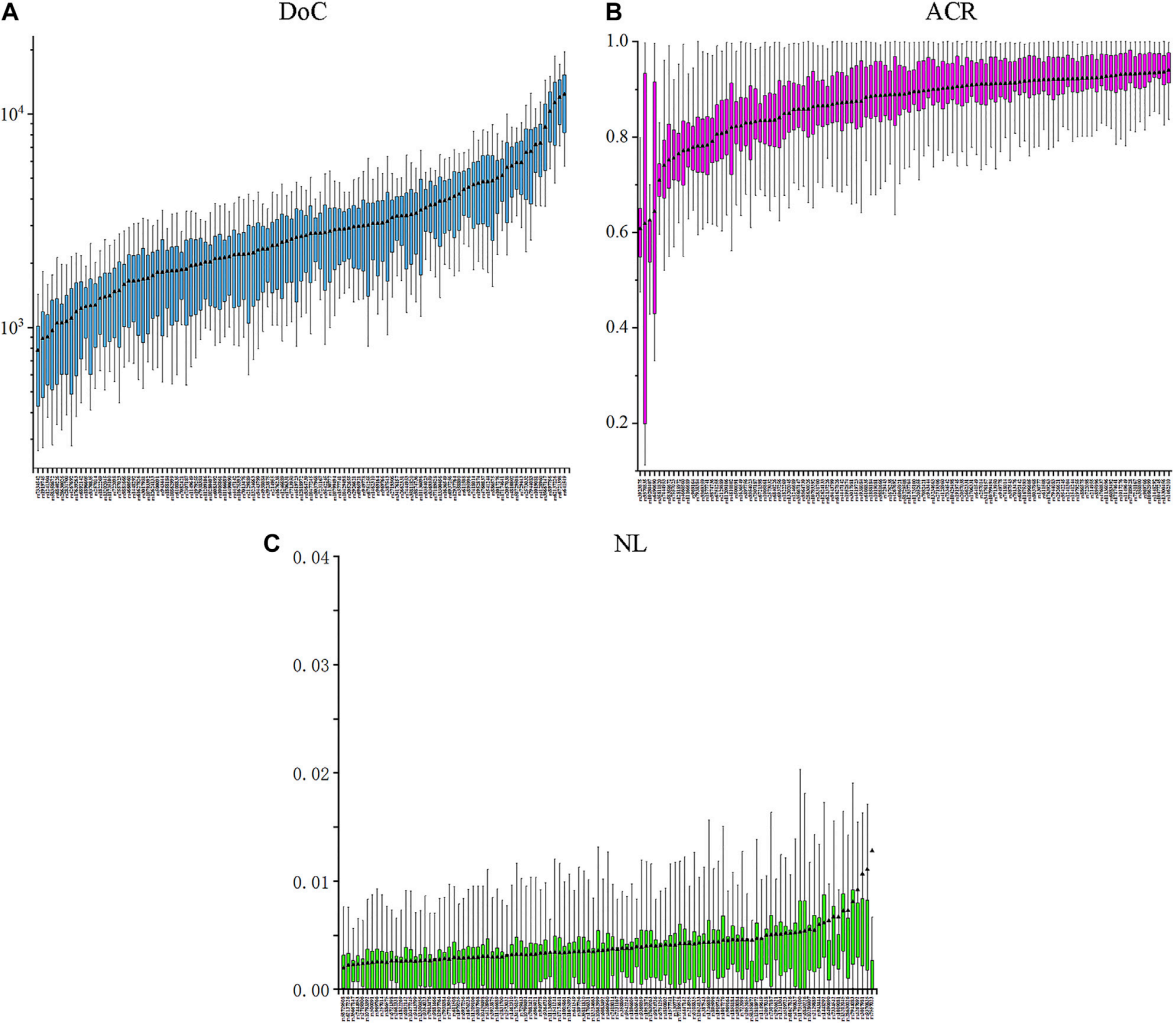
Box plots of the DoC **(A)**, ACR **(B)**, and NL **(C)** values based on the 111 II-SNPs in the IMM group.

The results of 20,757 genotyping data were detected at 111 II-SNPs in 187 IMM individuals. The specific DoC, ACR, and LB values of 111 II-SNPs in the IMM group were shown in Figure 2 and Supplementary Table S3. The DoC values of 111 II-SNPs ranged from 781 ± 495× at rs7334542 to 12375 ± 6,174× at rs641549. The ACR and LB values of 111 II-SNPs ranged from 0.6074 (rs3923875) to 0.9402 (rs1462310), and 0.0020 (rs58729508) to 0.0127 (rs7557023), respectively. The ACRs of four SNPs were between 0.6 and 0.7, including rs10280407 (0.6251), rs3923875 (0.6074), rs6490690 (0.6435), and rs9370835 (0.6181). Additionally, the average DoC, ACR, and LB values were calculated to be 5388×, 0.8672, and 0.0041, respectively.

### Allelic frequencies and forensic parameters of 111 II-SNPs in the IMM group

**FIGURE 3:**
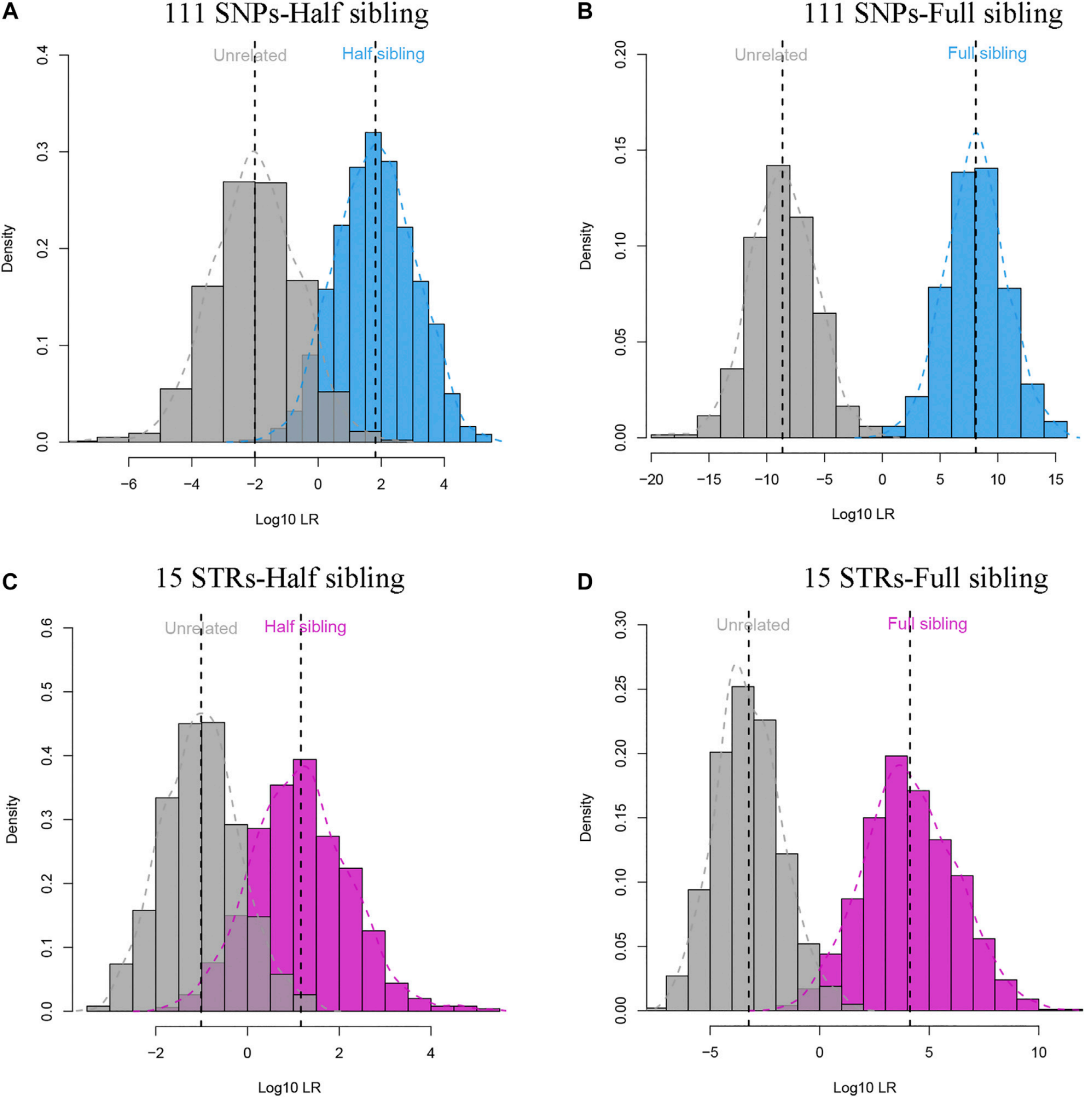
Log_10_LR distribution plots of full sibling and half sibling tests based on 111 II-SNPs and 15 STRs in the Manchu group. **(A)** Log_10_LR distributions to distinguish half siblings from unrelated individuals in the IMM group based on 111 II-SNPs; **(B)** Log_10_LR distributions to distinguish full siblings from unrelated individuals in the IMM group based on the 111 II-SNPs; **(C)** Log_10_LR distributions to distinguish half siblings from unrelated individuals in the Manchu group based on 15 STRs; **(D)** Log_10_LR distributions to distinguish full siblings from unrelated individuals in the Manchu group based on 15 STRs.

The 111 II-SNPs conformed to HWE (*p* > 0.05/111), and all pairwise SNPs were in linkage equilibrium (*p* > 0.05/6105) after Bonferroni correction. The results of LD tests at 111 II-SNPs are revealed in Supplementary Table S4. As illustrated in Supplementary Figure S1, the allelic frequencies of bi-allelic SNPs and multi-allelic SNPs of the 111 II-SNPs were in the range from 0.2941 to 0.7059, and 0.0080 to 0.6283, respectively. The allele frequency distributions of most bi-allelic SNPs were from 0.4 to 0.6. In Supplementary Figure S3 and Supplementary Table S5, the forensic parameters including PIC, Ho, He, PM, PD, and PE values of the 111 II-SNPs ranged from 0.3290 (rs11136096) to 0.5813 (rs943444), 0.4011 (rs77189528) to 0.7005 (rs28630026), 0.4163 (rs11136096) to 0.6576 (rs943444), 0.1975 (rs943444) to 0.4380 (rs969164), 0.5620 (rs969164) to 0.8025 (rs943444), and 0.1146 (rs77189528) to 0.4291 (rs28630026), respectively. Average PE, PIC, He, Ho, and PD values of the 111 II-SNPs were 0.2081, 0.3978, 0.5110, 0.5176, and 0.6397, respectively. Among 111 II-SNPs, the rs943444 locus exhibited the highest PIC, He, and PD values, and the rs28630026 locus exhibited the highest Ho and PE values. The cumulative PM, PD, and PE values of 111 II-SNPs in the IMM group were calculated to be 7.47E^-51^, 1-7.47E^-51^, and 1-4.17E^-12^, respectively. In Figure 3, full sibling and half sibling tests were simulated in the IMM group based on 111 II-SNPs. Significant LR distribution differences could be observed between full siblings and unrelated individuals in full sibling tests (Figure 3A) while a small proportion of LRs overlapped between half siblings and unrelated individuals in half sibling tests (Figure 3B). A 15-STR dataset from the Manchu group (Zhan et al., 2018) was simultaneously simulated (Figures 3C,D).

### Distributions of reference allelic frequencies based on 111 II-SNPs in the IMM group and 26 reference populations

The heatmap of reference allelic frequencies based on the 111 II-SNPs in the IMM group and 26 reference populations was shown in Supplementary Figure S2. The reference allelic frequencies of most bi-allelic SNPs displayed relatively balanced distributions (0.4-0.6) in East Asian populations. Several loci with low (less than 0.4) or high (greater than 0.6) reference allelic frequencies could be observed in multi-allelic SNPs in East Asian populations. Besides, the cluster analyses indicated that the IMM group first clustered with the CHB population and then clustered with other East Asian populations (JPT, CDX, KHV, and CHS).

### PCA of 111 II-SNPs in different continental populations

**FIGURE 4:**
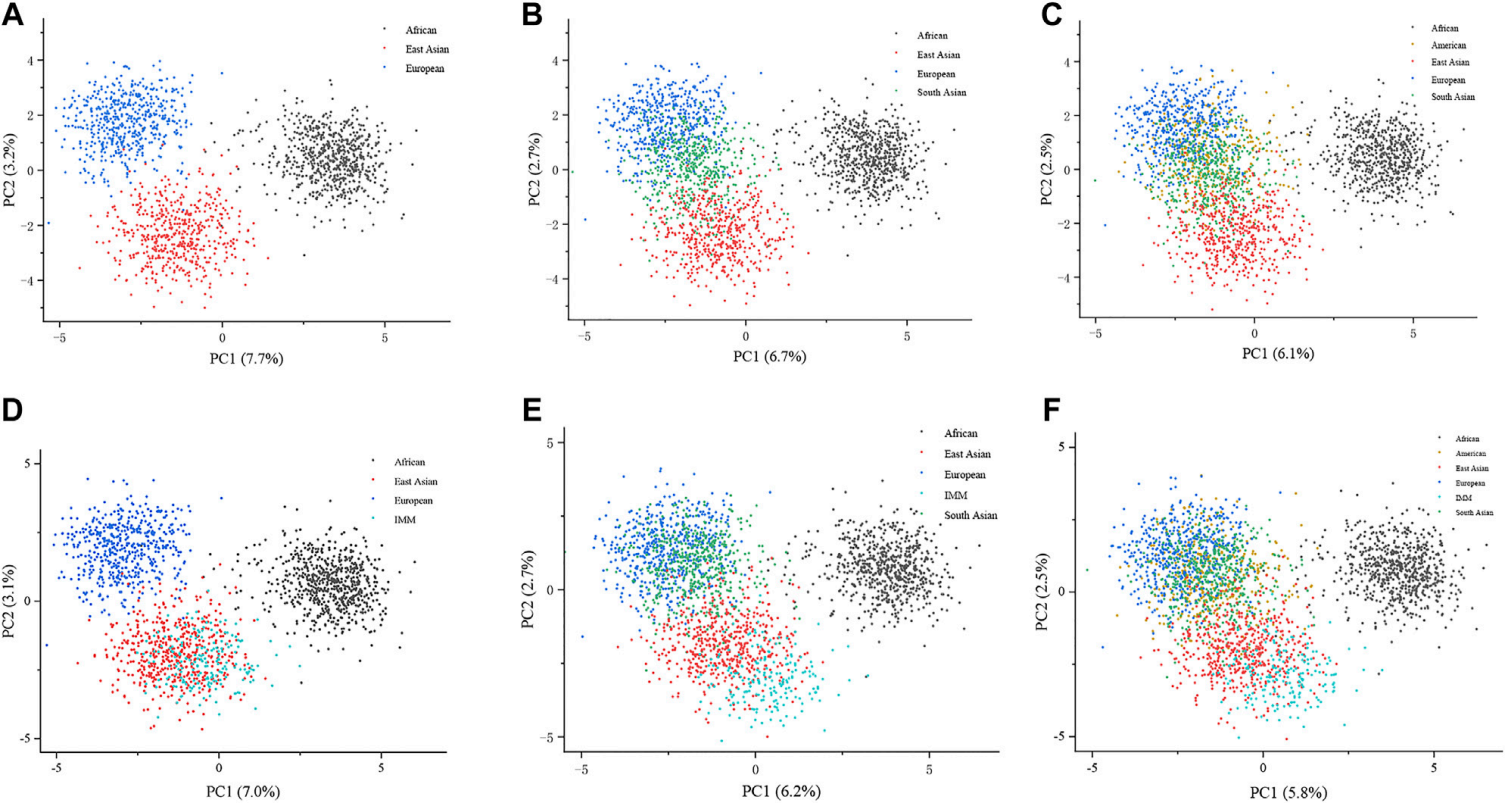
PCA plots at the individual level based on the 111 II-SNPs among the IMM group and 26 reference populations. **(A)** A PCA plot of African, East Asian, and European populations at the individual level based on the 111 II-SNPs. **(B)** A PCA plot of African, East Asian, and European populations plus IMM group at the individual level based on 111 II-SNPs. **(C)** A PCA plot of African, East Asian, South Asian, and European populations at the individual level based on the 111 II-SNPs. **(D)** A PCA plot of African, East Asian, South Asian, and European populations plus IMM group at the individual level based on the 111 II-SNPs. **(E)** A PCA plot of African, East Asian, South Asian, American, and European populations at the individual level based on the 111 II-SNPs. **(F)** a PCA plot of African, East Asian, South Asian, American, and European populations plus IMM group at the individual level based on the 111 II-SNPs.

To explore the genetic background of the IMM group, we plotted PCA at the individual level based on genotypes of the IMM group and 26 reference populations. Dots with different colors represent the individuals from different continents. As shown in Figure 4A, three continental individuals from Africa, Asia, and Europe were separated on different PC levels. However, with the addition of American and South Asian individuals, the two continental individuals mostly overlapped with European individuals on the upper left of the plot (Figures 4B,C). Individuals from the IMM group consistently clustered with East Asian individuals in Figures 4D–F, respectively.

### Population genetic distance comparisons based on 111 II-SNPs in different populations

**FIGURE 5:**
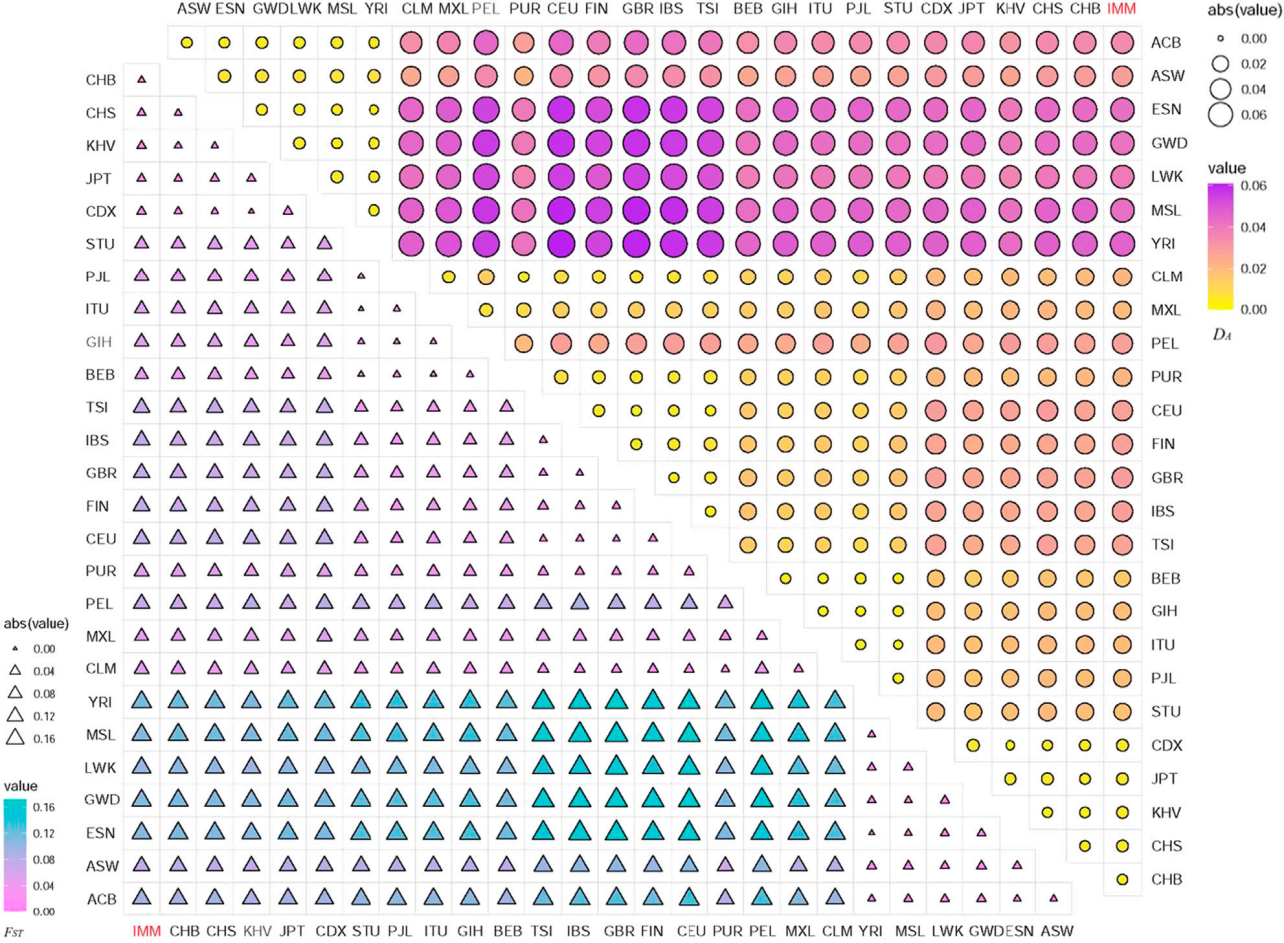
Heatmaps of the pairwise *D*_*A*_ and *F*_*ST*_ values among IMM group and 26 reference populations based on the 111 overlapped II-SNPs.

The *D*_*A*_ and *F*_*ST*_ values were utilized to estimate the genetic relationships among the IMM group and 26 reference populations via 111 II-SNPs. The heatmaps of *D*_*A*_ and *F*_*ST*_ values based on 111 II-SNPs among the IMM group and 26 reference populations were exhibited in Figure 5, respectively. The *D*_*A*_ and *F*_*ST*_ values were listed in Supplementary Table S6 and Supplementary Table S7, respectively. Minimal *D*_*A*_ (0.0029) and *F*_*ST*_ value (0.0038) were observed between the IMM group and the CHB population from East Asia. The maximal *D*_*A*_ (0.0463) and *F*_*ST*_ values (0.1163) were observed between the IMM group and the YRI population from Africa. The smaller *D*_*A*_ and *F*_*ST*_ values between the IMM group and East Asian populations ranged from 0.0029 (CHB) to 0.0052 (CDX), and 0.0038 (CHB) to 0.0112 (CDX), respectively. The larger *D*_*A*_ and *F*_*ST*_ values between the IMM group and the African populations ranged from 0.0284 (ASW) to 0.0463 (YRI), and 0.0732 (ASW) to 0.1163 (YRI), respectively. As shown in the heatmaps, populations from the same continent had relatively closer genetic distances and few genetic differentiations, while the opposite situation could be observed among populations from different continents.

### Phylogenetic tree construction based on 111 II-SNPs in the IMM group and 26 reference populations

**FIGURE 6:**
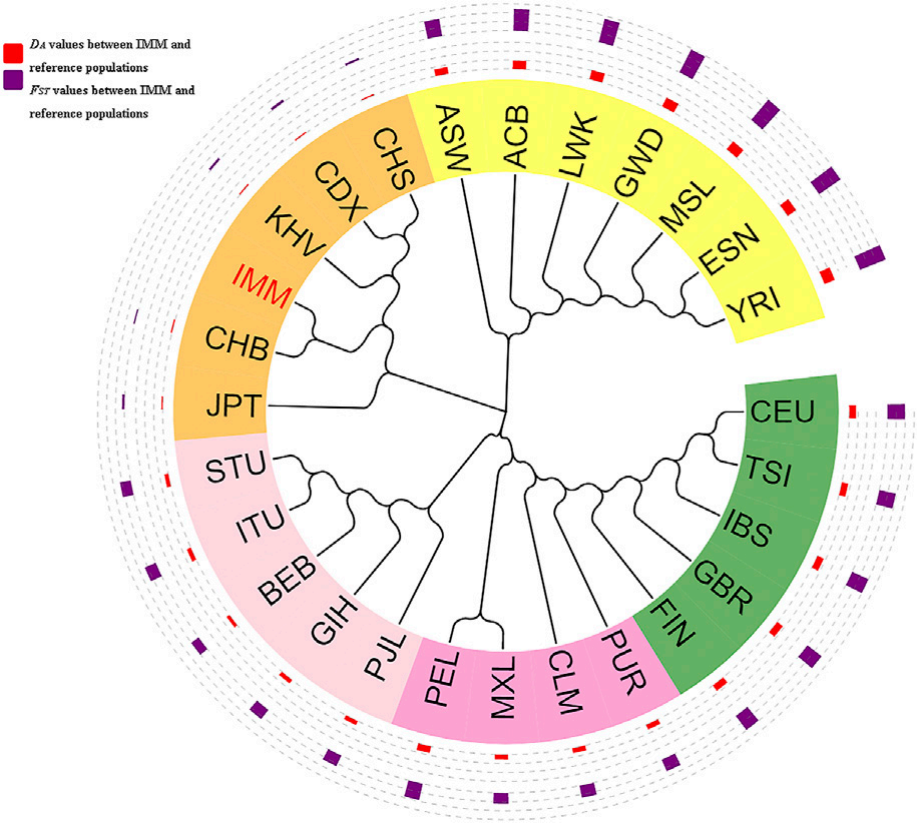
Phylogenetic tree based on the pairwise *D*_*A*_ values among the IMM group and reference populations on basis of 111 II-SNPs.

As shown in Figure 6, a phylogenetic tree was constructed based on *D*_*A*_ values among the IMM group and 26 reference populations. Different continents were represented by different colors. The orange, yellow, light pink, dark pink, and dark green identifiers represented East Asia, Africa, South Asia, America, and Europe, and the IMM group was highlighted in red. The red outer circle represented the *D*_*A*_ distance values between the IMM group and 26 reference populations, whereas the blue outer circle represented the *F*_*ST*_ values between the IMM group and 26 reference populations, and the height of the bar chart represented the magnitudes of *D*_*A*_ distances or *F*_*ST*_ values. The phylogenetic tree was mainly divided into two branches; one branch consisted of seven populations from Africa including ASW, ACB, LWK, GWD, MSL, ESN and YRI, and the other branch consisted of the remaining populations. Specifically, five populations (CEU, TSI, IBS, GBR, and FIN) in Europe, five populations (PJL, GIH, BEB, ITU, and STU) in South Asia, and six populations (IMM, JPT, CHB, KHV, CDX, and CHS) in East Asia, clustered in their sub-branches, respectively; and then with the four populations (PUR, CLM, MXL, and PEL) from America converged into one branch. The IMM group first aggregated with the CHB population, and then merged into the East Asia sub-branch. Phylogenetic analysis results indicated that the genetic relationships between the IMM group and East Asian population were closer, and the genetic relationships between the IMM group and African populations were further.

### Ancestry component inferences based on 111 II-SNPs in the IMM group and reference populations

**FIGURE 7:**
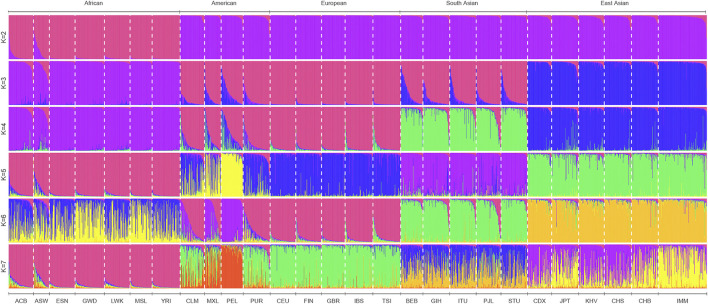
STRUCTURE analyses of 27 populations at *K* = 2-7 based on the 111 II-SNPs.

To infer the possible ancestral component proportion in the IMM group based on 111 II-SNPs, the population genetic structure analyses of the IMM group and 26 reference populations were conducted using STRUCTURE V2.3.4 software. We sorted the ancestral proportions from the lowest to the highest values by the preset ancestral information components (*K* = 2-7) in Figure 7. When *K* = 2, the 27 populations were mainly divided into African populations whose main ancestral information component was magenta and the non-African populations whose main ancestral information component was purple. The optimal preset *K* value was 3, which was assessed by the Structure Harvester program. East Asian populations represented as blue ancestor information component could be distinguished from non-African populations, while other populations were occupied by the high European ancestor information component. With the increase of *K* values, the South Asian populations were mainly occupied by the green ancestral component, and the American populations (PEL and MXL) were mainly occupied by the yellow ancestral component, which could be distinguished from the European populations when *K* = 4 and *K* = 5, respectively.

## Discussion

### Preliminary evaluation and validation of forensic efficacy of selected 111 II-SNPs

Due to the particularity of forensic research objects, the samples collected from the crime scene may have problems such as low quality and high degradation, so obtaining more clues from limited samples in a short period is conducive to improving the efficiency of cases handling. With an increase in the study of populations, some loci in the existing SNP kits showed low polymorphisms in the East Asian populations (Zhang et al., 2015). Moreover, in the previous research on SNP detection based on the SNaPshot technology, the number of SNPs available for simultaneous detection was limited, and cross-contamination occurred easily during PCR amplification (Daniel et al., 2015). With the enrichment of SNP information in the human genome and the upgrade in the detection technology of genetic markers, the simultaneous detection of multiple SNP genetic markers by MPS technology could well meet the needs of analyzing the degraded samples in forensic individual identification.

Therefore, to select the SNP genetic markers with relatively higher polymorphisms in East Asian populations and meet the needs of forensic individual identification, especially for degraded biological samples. The highly polymorphic SNPs in the East Asian populations were screened out based on the 1000 Genomes Project Phase III. Then, we conducted the preliminary assessment for 111 II-SNPs in East Asian populations; the Ho, PIC, and PD values of autosomal 111 II-SNPs in the East Asian populations were all greater than 0.4742, 0.3654, and 0.5838, respectively, and the cumulative PD value was higher than 1-1.08 × 10^-32^, which indicated that these autosomal 111 II-SNPs showed higher forensic application potential than the 94 SNPs reported previously (Wendt et al., 2016).

Subsequently, 187 individuals from the IMM group were collected to further evaluate the sequencing performances and genetic polymorphisms of 111 II-SNPs based on the MPS technique. The lowest DoC value was 781 ± 495× at the rs7334542 locus, according to the suggestion of the previous literature, which demonstrated that the DoC value was related to the sequencing methods, SNP typing algorithms, and the settings of sequencing analysis parameters. The DoC threshold was greater than 20× in the forensic application according to a previous report (Eduardoff et al., 2016). The sequencing depth of the allele of this SNP in 187 samples was greater than 20 reads, so this SNP locus could be used for subsequent statistical analyses. The mean ACR values of 111 II-SNPs were all greater than 0.7092, which was greater than the previously reported reference values suggested by some researchers (0.66) (Gill et al., 2000; Hansson et al., 2017). The results indicated that the allelic amplification efficiencies of most loci in this study were relatively balanced. The NL values of 111 II-SNP loci were close to zero, indicating that most loci had a lower level of background noise (Buchard et al., 2016). The DoC, ACR, and NL values showed that the sequencing performance was sufficient for reliable MPS-SNP genotyping.

The cumulative PD value (1-7.47E^-51^) of 111 II-SNPs was greater than those of the 15 STRs (0.9999999999999999849) and 19 STRs (0.9999999999999999999942) in the Manchu group reported by He and Liu (He and Guo, 2013; Liu et al., 2013) and the cumulative PE value (1-4.17E^-12^) of 111 II-SNPs was greater than those of the 90 SNPs (0.999999386152271, 0.999999607712827, and 0.999999696360182) from the Precision Identity Panel in Tibetan, Uygur, and Hui groups (Liu et al., 2018) and 110 SNPs (0.999999999862) and 119 SNPs (1-2.85E^-10^) in East Asian populations (Huang et al., 2018; Miao et al., 2021). The cumulative PM (7.47E^-51^) of 111 II-SNPs was less than those of the 94 SNPs (1.03E^-35^) from the ForenSeq™ DNA Signature Prep kit in Chinese population (Churchill et al., 2017). The cumulative PD, PE, and PM values suggest that the abovementioned SNPs had higher forensic efficiency in the IMM group. In a word, the results of the sequencing data and forensic parameters indicated that the screened 111 II-SNPs performed well based on the MPS technique and could meet the requirements of individual identification and paternity testing in the IMM group.

### Population genetic differences between the IMM group and 26 reference populations based on selected 111 II-SNPs

Due to China’s vast territory and numerous ethnic minorities, genetic differences occurred among different groups or the same group from different regions. Therefore, it is of great significance to comprehensively explore the population’s genetic background and ancestral origin using various genetic markers and detection techniques in forensic science. As a minority nationality, the Manchu group once established the Jin Dynasty and Qing Dynasty in 1,115–1,234 and 1,644-1910, respectively (He and Guo, 2013). Under the influence of long historical process and social development, Manchu ancestors, who originated near the Changbai mountains in northeast China, constantly communicated with the surrounding Han, Mongolian, Hezhe, Xibe, and other ethnic groups through intermarriage (Zhang et al., 2021).

In 2011, Zhao et al. analyzed 14 mtDNA haplogroups of 47 individuals from the Manchu group in the Chinese Jilin province, which gave the evidence to support the integration of Manchu and northern Han Chinese groups after the southward migration of Manchu group (Zhao et al., 2011). Previous genetic research on the Manchu group mainly came from the limited forensic genetic markers (autosomal STR, X-chromosome STR, Y-chromosome STR, and mtDNA), but there are few reports on the SNP data of the Manchu group so far. Therefore, in this study, PCA, pairwise *D*_*A*_, *F*_*ST*_, phylogenetic tree, and STRUCTURE analyses were performed to explore the genetic relationships between the IMM group and 26 reference populations based on 111 II-SNPs.

The PCA is a statistical method in which multiple indicators are converted into a few unrelated comprehensive indicators by dimensionality reduction of data, and the most appropriate two or three combinations of indicators are retained. The results of PCA analysis reflected that most IMM individuals and East Asian individuals overlapped together, indicating closer genetic relationships between the IMM group and East Asian populations based on the PCA plot at individual level.

The *D*_*A*_ is a metric to assess the genetic distance between pairwise populations and *F*_*ST*_ value, namely, the fixation index of pairwise populations, measures the degree of genetic differentiation by the allelic frequency distributions among pairwise populations (Wright, 1978). The *D*_*A*_ and *F*_*ST*_ results indicated that the IMM group shared closer genetic relationships with the East Asian populations, whereas the genetic differences between the IMM group and African populations were greater. A phylogenetic tree is a cladistic classification method used to describe the genetic evolutionary relationship between population and their hypothetical common ancestor. The result of phylogenetic tree showed that the cluster distributions of populations were mainly related to their geographical locations, and populations from the identical continent were mostly located on the same branch.

In the analyses of population genetic structure, the ancestral information component from East Asian populations (96%) in the IMM group was significantly higher than those from African (1.5%) and European populations (2.5%) when *K* = 3. The proportions of the ancestral information components indicated that the IMM group was similar to the East Asian populations with a gradually increasing *K* value, which was consistent with the results of the previous research on the genetic relationship of the Manchu group from Jilin province by STR genetic markers (He and Guo, 2013).

## Conclusions

We systematically screened 111 relatively high polymorphic autosomal SNPs for individual identification in East Asian populations and comprehensively assessed the forensic efficiency in East Asian populations from the 1000 Genomes Project phase III and further evaluated the sequencing performances, genetic polymorphisms, and forensic application values in the IMM group. The 111 II-SNPs revealed good sequencing performances and high genetic polymorphisms, which could provide effective tools for individual identification and parentage testing in the IMM group. However, to increase the widespread applications of the selected SNPs, more East Asian populations should be included, and various genetic markers should also be accommodated in the MPS platform.

## Data Availability

The original contributions presented in the study are included in the article/Supplementary Material; further inquiries can be directed to the corresponding author.
