# Supplementary figures and images for: Systematic selections and forensic application evaluations of 111 individual identification SNPs in the Chinese Inner Mongolia Manchu group
Source: Front Genet. 2022 Sep 5;13:944580. doi: 10.3389/fgene.2022.944580 (PMC9483854; doi:10.3389/fgene.2022.944580)

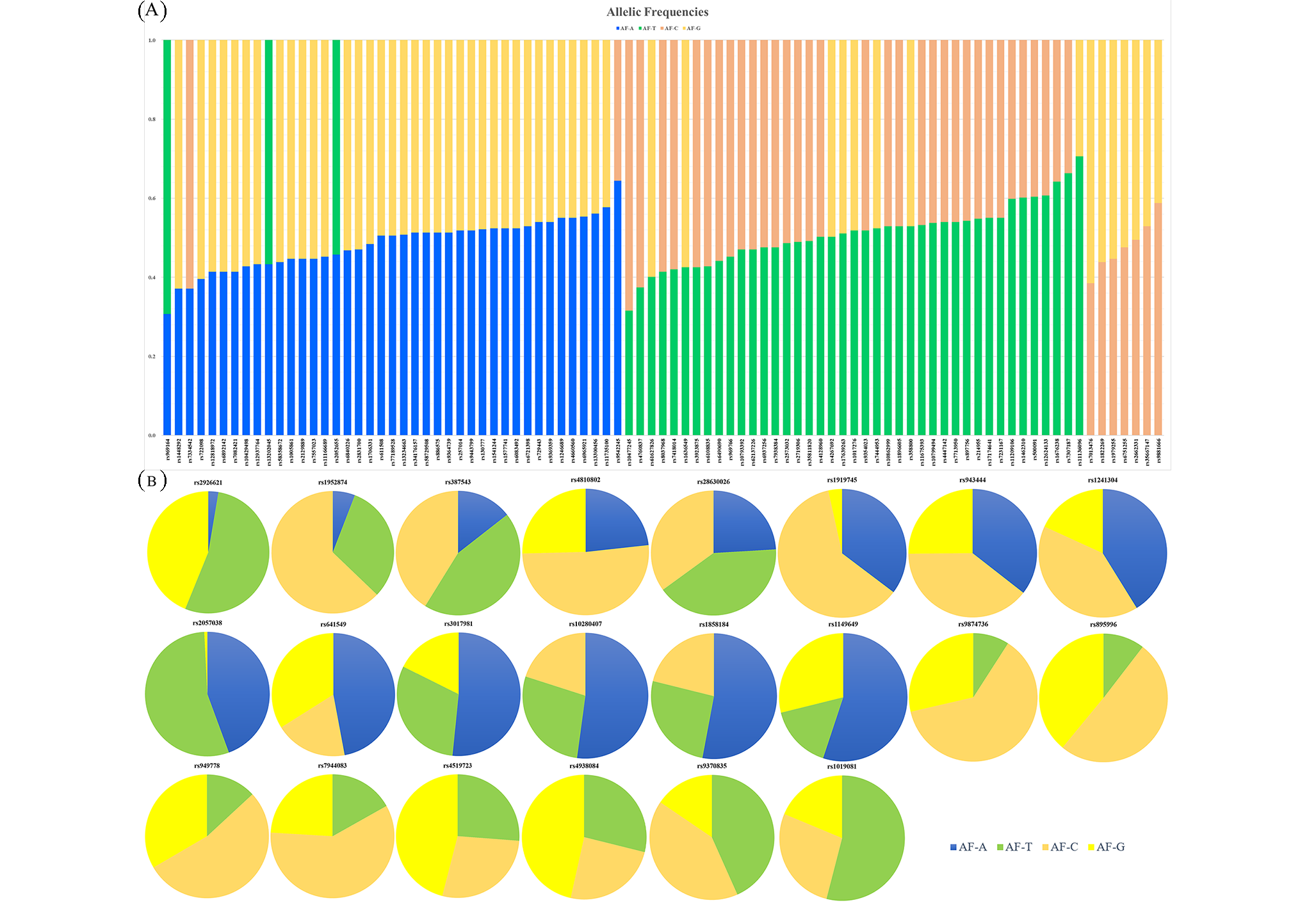

Supplement: Supplementary file 1 [file DataSheet1.zip › data sheet 1/Supplementary Figure 1. Bar plot of 89 diallelic SNPs (A) and pie plot of 22 multi-allelic SNPs (B) in the IMM group.tif]

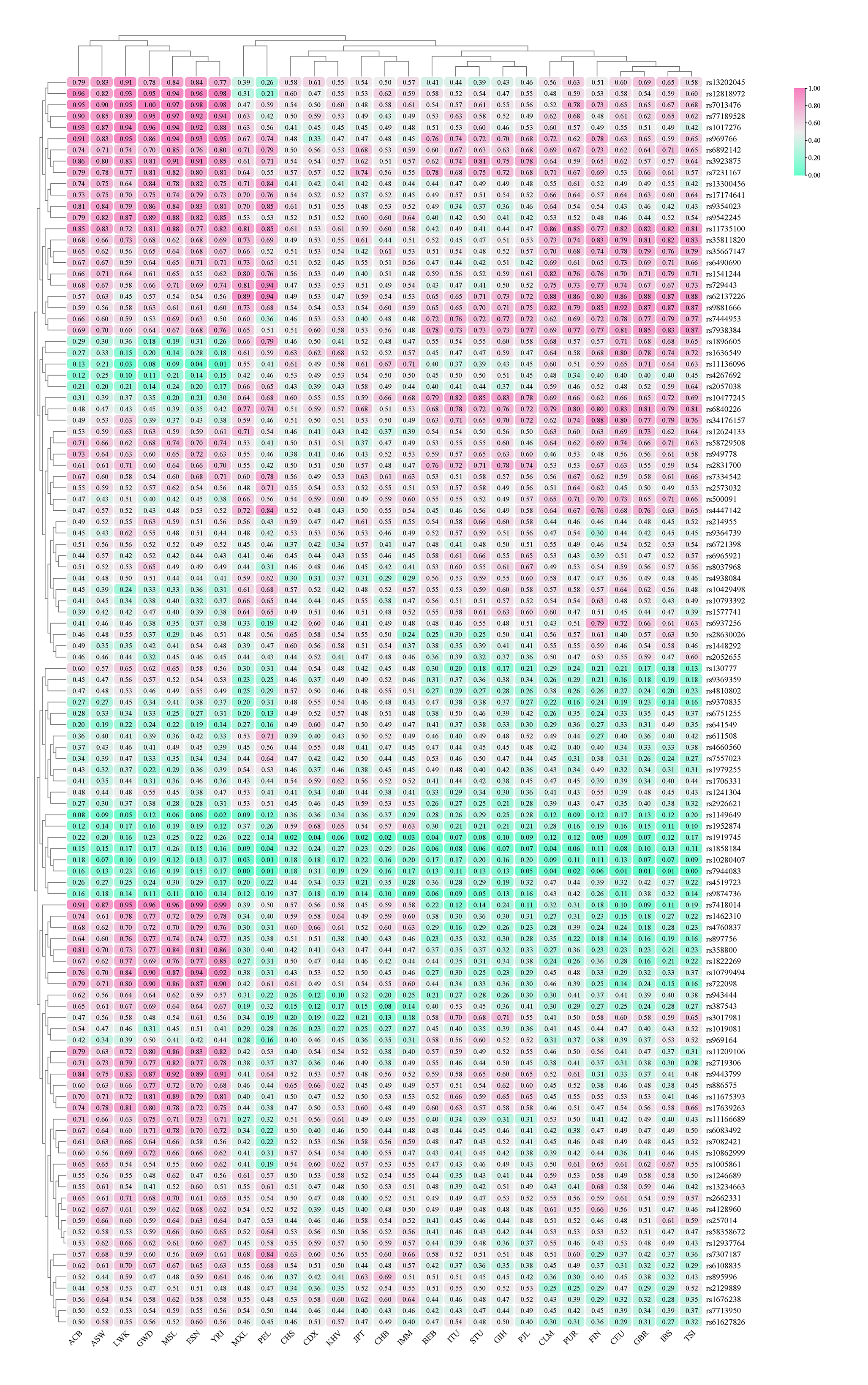

Supplement: Supplementary file 1 [file DataSheet1.zip › data sheet 1/Supplementary Figure 2. Heatmap of reference allelic frequencies based on the 111 II-SNPs among the IMM group and 26 reference populations.tif]

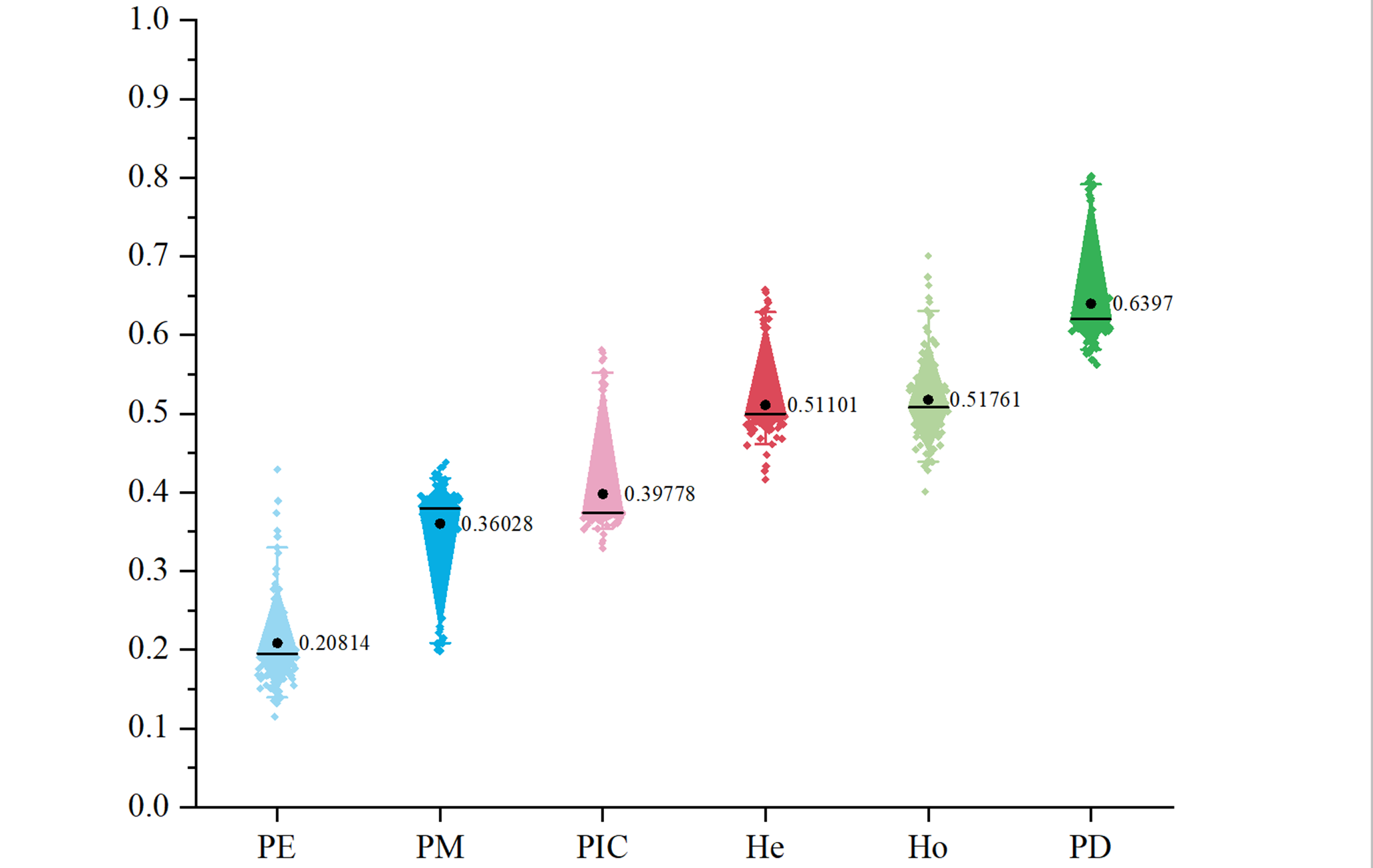

Supplement: Supplementary file 1 [file DataSheet1.zip › data sheet 1/Supplementary Figure 3. Box plots of forensic parameters based on the 111 II-SNPs in the IMM group.tif]
